# PD-L1 and c-MET expression and survival in patients with small cell lung cancer

**DOI:** 10.18632/oncotarget.9765

**Published:** 2017-06-01

**Authors:** Lulu Miao, Yunyun Lu, Yanjun Xu, Gu Zhang, Zhiyu Huang, Lei Gong, Yun Fan

**Affiliations:** ^1^ Department of Thoracic Medical Oncology, Hangzhou, 310022, Zhejiang, People's Republic of China; ^2^ Department of Pathology, Zhejiang Cancer Hospital, Hangzhou, 310022, Zhejiang, People's Republic of China

**Keywords:** small cell lung cancer, PD-L1, c-MET, expression, prognosis

## Abstract

**Background:**

Blocking the binding between the PD-1 and PD-L1 has been reported to produce antitumor responses. The MET/HGF axis appears to be another signaling pathway frequently altered in small cell lung cancer (SCLC). Our study was aimed to investigate the expression and prognostic roles of PD-L1 and c-MET in SCLC.

**Methods:**

The expression levels of PD-L1 and c-MET were evaluated by immunohistochemical analysis in 83 SCLC specimens. Survival analysis was performed using the Kaplan-Meier method.

**Results:**

Of the SCLC specimens, 51.8% and 25.3% exhibited positivity for PD-L1 and c-MET, respectively. Higher PD-L1 expression in tumor specimens was significantly correlated with a limited disease (LD) stage, normal levels of serum lactate dehydrogenase (LDH) and neuron-specific enolase (NSE). No association was found between the levels of c-MET and PD-L1 expression or between c-MET expression and other clinical characteristics. SCLC patients with PD-L1-positive tumors showed significantly longer overall survival (OS) than patients with PD-L1-negative tumors (17.0 vs 9.0, p=0.018). Conversely, those with positive c-MET expression exhibited a shorter OS trend (12.0 vs 15.0, p=0.186). However, sub-analysis of LD-stage patients revealed longer OS among the c-MET-negative group (25.0 vs 14.0; p=0.011). The OS of patients with positivity for both PD-L1 and c-MET showed no significant difference compared with other patients (p=0.17). According to multivariate analyses, neither PD-L1 nor c-MET immunoreactivity was a prognostic factor.

**Conclusion:**

Expression of PD-L1 was correlated with LD stage and might serve as a prognostic for better OS in SCLC patients. In LD-stage patients, high c-MET expression might be predictive of a poor outcome.

## INTRODUCTION

Small cell lung cancer (SCLC), which accounts for approximately 13-15% of all primary lung cancers [1], is one of the most aggressive types of lung cancer. Patients with SCLC are staged as limited disease (LD) or extensive disease (ED) based on the anatomical extent. Approximately 60%-70% of SCLC patients are staged at the ED stage at the time of diagnosis [2]. Although SCLC is highly sensitive to initial chemo- and radiation therapy, most patients inevitably suffer from early relapse and acquired drug resistance. The prognosis of SCLC patients remains very poor. For LD patients, the median overall survival (mOS) is 16–24 months, with a two-year survival rate of 25%. For ED patients, the mOS is 8–13 months, with a dismal two-year survival rate of approximately 5% [3]. Despite rapid progress in our knowledge of the molecular biology of non-small cell lung cancer (NCSLC) and the development of targeted therapy, conventional chemo- and radiation therapy for SCLC over the last few decades has remained largely unchanged [4]. Thus, to improve the outcome of SCLC patients, it is urgent to explore the underlying molecular mechanisms and immune-regulation in SCLC carcinogenesis.

As the aberrant activation of co-inhibitory pathways is a key determent of immune suppression, blocking immune checkpoints with monoclonal antibodies has recently emerged as a new therapeutic strategy in cancer treatment [5]. Programmed death 1 (PD-1), a receptor expressed on the surface of T cells, is involved in inhibitory signal transmission [6, 7]. Its ligand, programmed death–ligand 1 (PD-L1), is frequently overexpressed in many types of human cancer [8], inducing T cell impotence and ultimately achieving immune evasion. Recent clinical trials have shown the promising antitumor activity of PD-L1 and PD-1 antibody blockade in various malignancies, including NSCLC and SCLC [9–13]. Albeit rare, there are also reports on the promising efficacy of PD-L1 and PD-1 antibodies in SCLC patients, both PD-L1-positive and PD-L1-unselected populations [14–15]. Both PD-1 and PD-L1 are expressed on the surface of SCLC cells, though the biological implications and the exact functions of PD-1 and PD-L1 in SCLC remain unclear [16].

The MET/HGF axis appears to be another signaling pathway that when aberrant, is involved in SCLC invasiveness and progression [3]. Mesenchymal-epithelial transition (MET) factor receptor is activated upon binding its ligand hepatocyte growth factor (HGF), resulting in activation of different intracellular signaling pathways responsible for promoting proliferation and invasiveness. Furthermore, increasing evidence indicates that the activated MET pathway is a prognostic factor for a poor outcome in lung cancer and many other solid tumors [17–20]. Regardless, there are limited data on the prevalence and prognostic role of MET expression in SCLC.

In the present study, we aimed to evaluate the expression levels of PD-L1 and MET as two potential therapeutic targets using IHC analysis of SCLC specimens and to further explore their clinical relevance and prognostic role in this aggressive malignancy.

## RESULTS

### Patient characteristics

The clinical characteristics of 83 SCLC patients were collected in our study (Table 1). The median age of the patients at diagnosis was 59 years (range 35-84 years). The majority of patients were males (72 cases, 86.7%). A total of 79 samples (95.2%) were obtained from primary lung lesions and four (4.8%) from metastatic sites, including lymph node metastasis in three (3.6%) and brain metastasis in one (1.2%). Among the specimens from lung tumors, 16 (19.3%) were from lung cancer resections and 55 (66.3%) from biopsy specimens by means of fiber bronchoscopy (47, 56.6%) or lung puncture (8, 9.6%). Thirty-six patients (43.4%) had ED-stage and 47 (56.6%) LD-stage disease at the time of diagnosis. Among the 47 LD-SCLC patients, 22 (46.8%) received concurrent or sequential chemoradiotherapy, 15 (31.9%) underwent surgical resection followed by chemotherapy, and 10 (21.3%) received single chemotherapy or radiotherapy. The ED-SCLC patients were treated with first-line chemotherapy, and one of them also received palliative resection. The serum LDH level was higher than the normal limit of 240 IU/L in 12 patients (14.5%), and 53 patients (63.9%) had abnormally increased NSE serum levels (higher than 17.0 ng/ml).

**Table 1:** Association between PD-L1 or c-MET expression and clinicopathological characteristics of SCLC patients

Variables	No. of patients	PD-L1 expression		p-value	c-MET expression		p-value
**Positive**	**Negative**	**Positive**	**Negative**
Age							
<70	75	40	35	0.902	20	55	0.235
≥70	8	3	5		1	7	
Sex							
Male	72	37	35	0.953	18	54	0.262
Female	11	6	6		3	8	
PS status							
0-1	80	42	38	0.229	20	60	0.763
2-3	3	1	2		1	2	
Stage							
LD	47	29	18	0.004	10	37	0.450
ED	36	14	22		11	25	
LDH							
Normal	71	40	31	0.031	17	54	0.972
Abnormal	12	3	9		4	8	
NSE							
Normal	30	21	9	0.005	6	24	0.824
Abnormal	53	22	32		15	38	

### PD-L1 and c-MET expression

**Figure 1:**
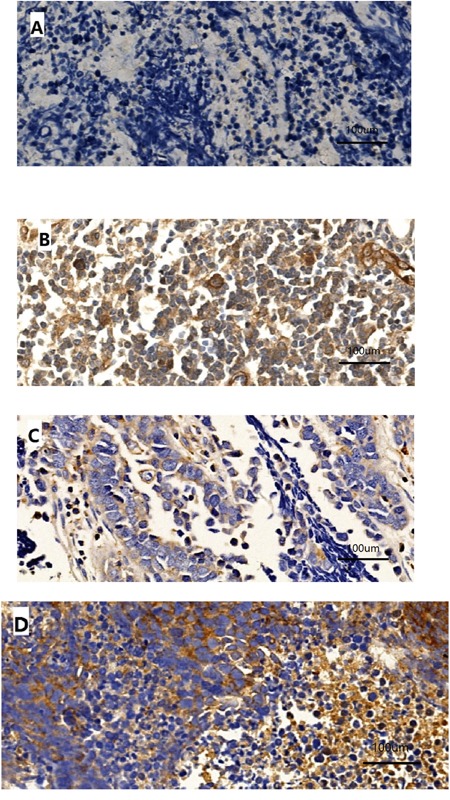
Representative patterns of PD-L1 immunostaining in SCLC tumors with negative A or strong positive **B**. staining intensity and c-MET immunostaining with negative **C**. or strong positive D. staining intensity. Original magnification, ×400.

Immunoreactivity for PD-L1 was observed in the membrane and/or cytoplasm of tumor cells and stromal lymphocytes (Figure 1). The median PD-L1 expression score was 15. Forty-three (51.8%) patients exhibited positive tumor staining for PD-L1. Immunostaining for c-Met was found in the cytoplasm of tumor cells and was positive in 21 cases (25.3%). The median c-MET expression score was 20. Among all 83 SCLC patients, 14 (16.9%) were found to express both PD-L1 and c-MET.

### Correlation between PD-L1 and patient characteristics

**Figure 2:**
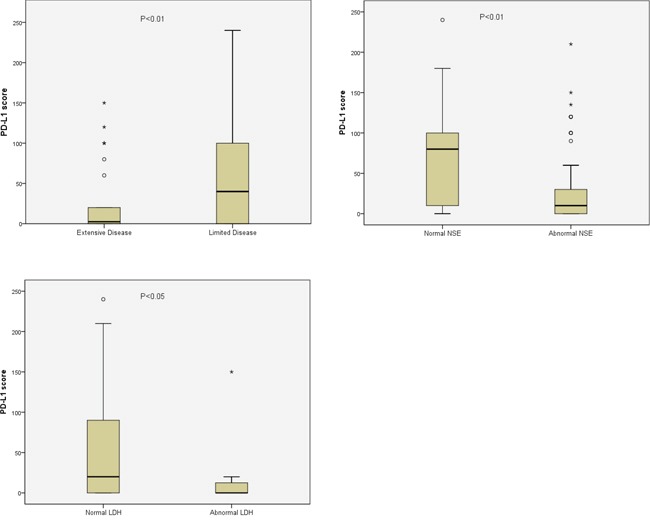
Significant association of PD-L1 staining score with disease stage, LDH level or NSE level Data are presented as box-and-whisker plots, and p values were determined using the Wilcoxon rank-sum test.

A higher expression level of PD-L1 in tumor specimens was significantly correlated with a limited disease (LD) stage (p=0.004), a normal serum LDH level (p=0.031), and a normal NSE level (p=0.005) (Figure 2). No association was found between the levels of PD-L1 and c-MET expression (p=0.082).

### Correlation between c-MET and patient characteristics

No significant correlation was observed between c-MET expression and disease stage (LD vs ED, p=0.450), serum LDH level (normal vs abnormal, p=0.972), serum NSE level (normal vs abnormal, p=0.824), age (<70 vs ≥70, p=0.235), sex (male vs female, p=0.262) or other clinical characteristics. The relationship between PD-L1 or c-MET expression and patient demographics is shown in Table 1.

### Survival analysis

**Figure 3:**
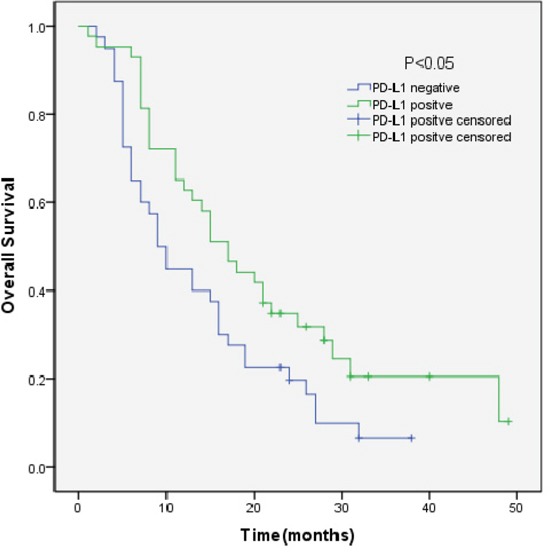
Kaplan–Meier curves of overall survival (OS) in PD-L1-positive vs PD-L1-negative patients Overall survival of 83 SCLC patients in relation to PD-L1 status.

**Figure 4:**
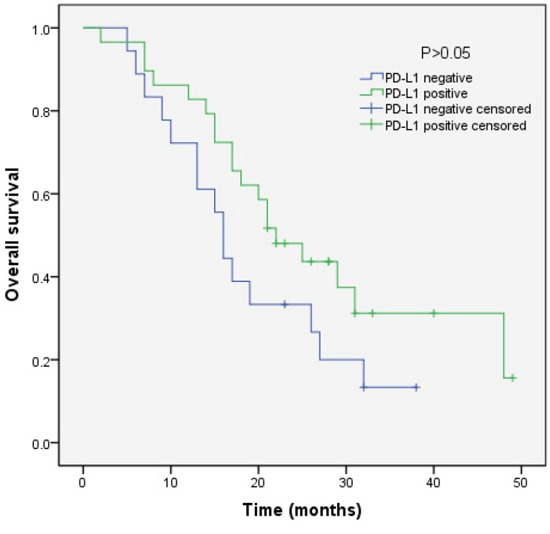
Kaplan–Meier curves of overall survival (OS) in PD-L1-positive vs PD-L1-negative patients with LD-stage disease Overall survival of 47 SCLC patients with LD-stage disease in relation to PD-L1 status.

**Figure 5:**
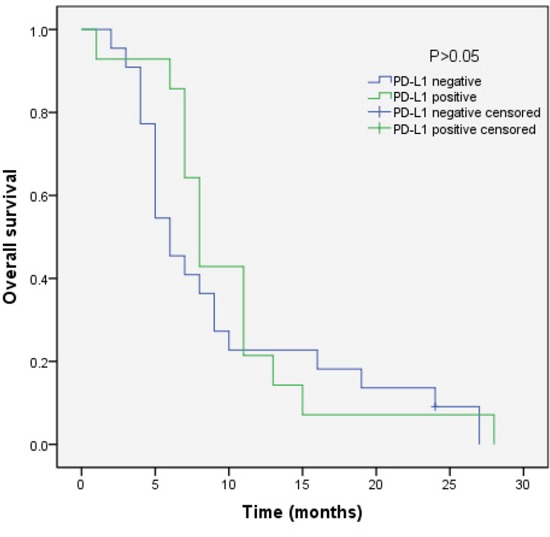
Kaplan–Meier curves of overall survival (OS) in PD-L1-positive vs PD-L1-negative patients with ED-stage disease Overall survival of 36 SCLC patients with ED-stage disease in relation to PD-L1 status.

**Figure 6:**
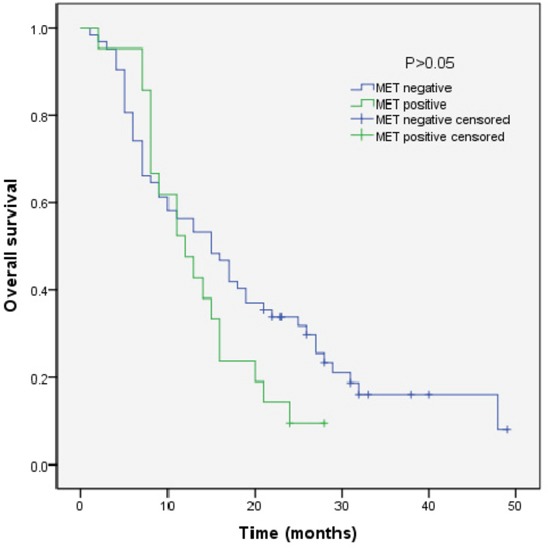
Kaplan–Meier curves of overall survival (OS) in MET-positive vs -negative patients Overall survival of 83 SCLC patients in relation to c-MET status.

**Figure 7:**
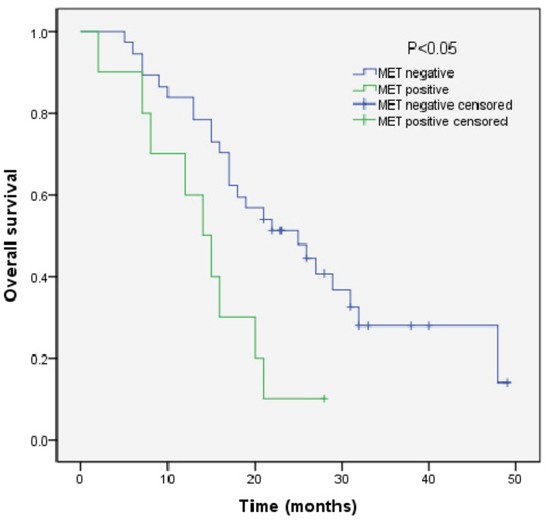
Kaplan–Meier curves of overall survival (OS) in MET-positive vs -negative patients with LD-stage disease Overall survival of 47 SCLC patients with LD-stage disease in relation to c-MET status.

We analyzed the outcomes of patients according to the immunohistochemical status of PD-L1 and c-MET. SCLC patients with PD-L1-positive tumors showed significantly longer overall survival (OS) than patients with PD-L1-negative tumors (median OS, 17.0 vs 9.0, p=0.018) (Figure 3). Sub-analysis of the LD-SCLC patients showed no significant difference in OS between the PD-L1-positive and -negative groups (22.0 vs 16.0, p=0.10) (Figure 4). Similarly, no significant difference in OS was found for the ED-SCLC patients (8.0 vs 6.0, p=0.29) (Figure 5). Those SCLC patients with positive c-MET expression exhibited a trend of shorter OS (12.0 vs 15.0, p=0.186), but the difference was not significant (Figure 6). However, sub-analysis of the LD-stage patients revealed a longer OS for the c-MET-negative group (25.0 vs 14.0; p=0.011) (Figure 7). The OS of patients showing positivity for both PD-L1 and c-MET was not significantly different from that of the other patients (p=0.17). Multivariate analyses revealed that LD stage and good performance status, but not PD-L1 or c-MET immunoreactivity, were independently predictive of better OS (Table 2).

**Table 2:** Multivariate analysis factors for overall survival

Factor	Hazard ratio	95% CI	p-value
PS (0-1/2-3)	2.808	1.111-7.098	0.029
Stage (LD/ED)	3.364	1.901-5.953	<0.001
NSE level (Low/High)	1.673	0.925-3.027	0.089
LDH level (Normal/Abnormal)	1.220	0.580-2.568	0.600
PD-L1 expression (Positive/Negative)	0.943	0.572-1.557	0.820
c-MET expression (Positive/Negative)	0.732	0.401-1.333	0.307

## DISCUSSION

Antibody-induced blockade of PD-L1 has resulted in durable tumor regression and prolonged disease stabilization in patients with advanced cancers, including NSCLC, melanoma, and renal-cell cancer [11]. Indeed, the success of blocking co-inhibitory pathways in many malignant neoplasms has inspired more confidence and enthusiasm in antitumor therapy. However, clinical research on the relationship between PD-L1 and SCLC is rare. Several studies have shown that PD-L1 expression may serve as a prognostic factor. PD-L1 expression by tumors has also been theorized to be a potential biomarker for patients who may have higher response rates to PD-1 pathway-targeting agents. However, the results to date are contradictory. MET, another crucial process that represents one of the most important mechanisms of progression and invasiveness [21–22], is also a potential therapeutic target. In contrast to the strong evidence for the involvement of MET in NSCLC, limited data are available for SCLC and MET. Given the insufficient treatment options in SCLC and its poor prognosis, we decided to examine the expression of PD-L1 and c-MET and their clinical correlation in SCLC.

Our data showed that high levels of PL-L1 expression were correlated with LD stage and normal levels of LDH and NSE. These results were consistent with Hidenobu's study including 102 SCLC patients [23]. Survival analysis also showed that SCLC patients with PD-L1-positive tumors had significantly longer OS. However, our multivariate analyses revealed that PD-L1 immunoreactivity was not predictive of better OS. Previous results on the prognostic role of PD-L1 expression are also controversial. Some reports have shown PD-L1 protein expression to be associated with better prognosis in patients with lung cancer, colorectal cancer, breast cancer, and malignant melanoma [23–29]. In contrast, other studies have reported that PD-L1 expression is associated with poor prognosis in patients with NSCLC, gastric carcinoma, hepatocellular carcinoma and pancreatic cancer [30–33]. There are several possible explanations for the conflicting results. First, the human immune system is a dynamic, precisely regulated, multi-step process, and PD-L1 is an inducible marker that can be upregulated or downregulated over time [34]. Second, determination of PD-1 expression is generally performed via immunohistochemistry, using various antibodies in different malignancies. Third, the threshold for PD-L1 positivity differed among previous studies. In our study, we defined positive PD-L1 expression as staining in more than 5% of tumor cells, the same criteria used in clinical trials [12]. The optimal threshold for PD-L1 positivity in terms of prognosis discrimination remains undefined and deserves further investigation in future clinical trials.

The results of our study showed that expression of c-MET was relatively less common, in only 25.3% of SCLC specimens, and no statistically significant correlations were observed between c-MET expression and disease stage or other clinical characteristics or survival outcome. However, sub-analysis of LD-stage patients showed a longer OS for the c-MET-negative group. Although several clinical studies in patients with NSCLC have demonstrated that MET overexpression is associated with a poor survival rate [35–38], in our study, the prognostic significance of MET overexpression was only found in SCLC LD-stage patients. The accuracy of the results may be influenced by the immunohistochemical methodology employed. Further research is needed to clarify the prognostic role of the MET signaling pathway using different methods, such as fluorescence in situ hybridization (FISH) and gene sequencing.

Previous studies have shown that PD-L1 expression is driven by various oncogenic signaling pathways. For example, Azuma found that high PD-L1 expression is associated with the presence of EGFR mutations in surgically resected NSCLC [39]. Tang Y also reported that PD-L1 tended to be associated with mutant EGFR (p=0.067) [40]. However, we did not find an association between PD-L1 expression and c-MET expression in our study. Further research is needed to elucidate the relation between PD-L1 expression and the MET signaling pathway.

Our study also had certain other limitations. First, the number of patients enrolled was relatively small. Second, as 47 (56.6%) of the patients were LD-stage patients, the representativeness of the sample may not be adequate. Third, the therapies received by these patients were not standardized and might have affected survival outcomes. We expect that future large-sample studies, which could balance all relevant clinical factors, may further verify our findings.

In conclusion, we evaluated the expression of PD-L1 and c-MET in patients with SCLC and found that PD-L1 expression might be prognostic for a better OS. High expression levels of c-MET revealed a trend of worse outcomes associated with poor prognosis in LD-stage patients. Our results revealed the clinical relevance and potential prognostic roles of these two factors, providing a foundation for further research on immunotherapy or targeted therapy for SCLC.

## PATIENTS AND METHODS

### Patients

We screened 158 patients diagnosed with SCLC at Zhejiang Cancer Hospital between July 2010 and December 2012. For immunohistochemistry analysis, we excluded 75 patients diagnosed using cytological specimens or whose tissue blocks contained too few tumor cells to allow the assay. Eighty-three of these patients who had adequate paraffin-embedded tumor specimens for immunohistochemical detection and with complete clinical and follow-up data were enrolled. This study was conducted in accordance with the provisions of the Declaration of Helsinki and was approved by the Institutional Review Board of Zhejiang Cancer Hospital.

### Immunohistochemical analysis

Paraffin-embedded tumor tissue was sectioned at a thickness of 4 μm, and the sections were mounted on glass slides for immunohistochemical analysis of PD-L1 and c-MET with the use of the BenchMark XT platform (Ventana Automated Systems, Tucson, AZ). Standard indirect immunoperoxidase procedures were used for IHC. Briefly, slides were dewaxed and rehydrated in distilled water. Endogenous peroxidase activity was blocked using 0.5% H_2_O_2_. The sections were treated with 10% normal goat serum (DakoCytomation; Dako, Carpinteria, CA, USA) for 20 minutes and incubated with primary antibodies against PD-L1 (anti-B7-H1/PD-L1/CD274 Antibody, clone SP66, SPRINGBIO, USA) and c-MET (ZA-0547, clone SP142, SPRINGBIO, USA) at room temperature. The sections were further incubated with a peroxidase-labeled secondary antibody (Polymer HRP Goat anti-Mouse & Rabbit IgG (Cat No. D22-110)) for 30 minutes at room temperature. For visualization of the antigen, the sections were immersed in 3-amino-9-ethylcarbazole plus substrate-chromogen (DakoCytomation) for 30 minutes and counterstained with Gill's hematoxylin.

Two well-experienced pathologists examined the immunohistochemical slides without any prior information on the clinicopathological features of the patient samples. The percentages of PD-L1- and c-MET-positive tumor cells and staining intensity were evaluated for each sample. The intensity of staining was evaluated according to the following scale: 0, no staining; 1, weak staining; 2, moderate staining; and 3, strong staining. A semi-quantitative approach was used to generate a score for each tissue core. The percentage of stained cells (0–100%) was multiplied by the dominant intensity pattern of staining, ranging from 0 to 3. Therefore, the overall semiquantitative score ranged from 0 to 300. In the absence of any standardized scoring system, tumors with PD-L1 staining in over 5% of the cells were scored as positive for PD-L1 expression, according to previous studies [8, 41–42]. Tumors with strong c-MET staining in at least 10% or weak to moderate staining in at least 40% of the cells were scored as positive for c-MET expression [43].

### Statistical analysis

Statistical analyses were performed using the SPSS analytical software (IBM). Clinical characteristics and their correlations with PD-L1 or c-MET expression were examined using the χ^2^ test or Fisher's exact test. Differences between the PD-L1 or c-MET median score were analyzed using the Wilcoxon rank-sum test. Overall survival (OS) was measured from the administration of treatment until the date of death or last follow-up. Survival curves were generated using the Kaplan–Meier method, and the log-rank test was used to assess the statistical significance of the differences between two groups. Further multivariate survival analysis was conducted using the Cox regression model. Statistical significance was defined as p<0.05.
